# Cyclodextrin Based Nanoparticles for Drug Delivery and Theranostics

**DOI:** 10.34172/apb.2020.022

**Published:** 2020-02-18

**Authors:** Dipak Dilip Gadade, Sanjay Sudhakar Pekamwar

**Affiliations:** ^1^Department of Pharmaceutics, Shri Bhagwan College of Pharmacy, CIDCO, N-6, Dr. Y.S. Khedkar Marg, Aurangabad-431001, India.; ^2^School of Pharmacy, SRTM University,Vishnupuri, Nanded- 431606, India.

**Keywords:** Cyclodextrin, Nanoparticle, Solubility, Theranotics, Stability, Controlled release

## Abstract

Colloidal nanoparticulate technology has been described in the literature as a versatile drug delivery system. But it possesses some inherent lacunae in their formulation. Cyclodextrins (CDs) have been extensively reported for the solubility enhancement of poorly water-soluble drugs. The CDs can cause intervention in aspects related to nanoparticles (NPs) that include improving drug loading in nano-system, improving stability, site-specific/targeted drug delivery, improving solubility profile and absorption of the drug in nanosystem with consequent improvement in bioavailability, with the possibility of controlled release, safety and efficacy. They find application in for simultaneous diagnosis and therapeutics for better treatment procedures. The current communication is focused on the application of CDs to overcome troubles in nanoparticulate formulation and enhancement of their performance. It also envisages the theranostic aspects of CDs.

## Introduction

The research and development of nanoscale systems are gaining popularity from the last few years due to their associated potential applications in the pharmaceutical and biotechnological field. Nanoparticles (NPs) are small colloidal particles made of biodegradable or non-biodegradable materials. The average diameter of NPs ranges from 10 to 1000 nm, but the particles of diameter <200 nm are often referred as nanomedicine.^1^ The drug is dissolved, dispersed, encapsulated, entrapped or attached to NP matrix.^2,3^ Depending on the method of preparation, nanospheres (the drug is uniformly dispersed in a matrix) or nanocapsules (the drug is confined in a cavity) are obtained which possess different properties and release characteristics suitable for best drug delivery or drug encapsulation.^4^

Due to the small size of NPs they can penetrate tissues and small capillaries offering advantages including long circulation time, improvements in the target to non-target concentration ratio, increased residence at target site and improved cellular uptake.^5^ This is achieved by opsonization of NPs followed by macrophage activation in circulation. The surface area per unit mass of NPs compared with other multi-particulate systems is the unique characteristic feature which adds up to their functionality. This may be useful in improving the aqueous solubility of a drug candidate, which is an important physicochemical factor further affecting its dissolution and bioavailability.^6^

Various types of nanoparticulate systems were developed for drug delivery, including polymeric NPs^7,8^ lipid NPs (viz. first generation-solid lipid NPs and second generation-nanostructured lipid carriers),^9^ polymeric micelles, liposome, nanotubes, nanocrystals, dendrimer, metallic NPs,^10^ quantum dots and magnetic NPs.^11^

NPs are employed for diverse applications, including site-specific and targeted drug delivery in cancer,^12^ as the leaky and defective architecture of tumour allows interstitial access to NPs which is popularly known as enhanced permeation and retention.^13,14^ These are explored for drug delivery through different routes of administration including oral,^15^ pulmonary,^16^ nasal, parenteral,^17^ ocular,^18^ brain^19^ and dermal-transdermal routes.^20,21^

The reports focused on cyclodextrin (CD) which discusses its role in oral cancer therapy,^22^ pharmaceutical and biomedical applications of CD-based nanogels^23^ and in drug and gene delivery are available.^24-27^ Despite the varied potential of NPs, they possess lacuna related to some physicochemical and pharmaceutical aspects. These challenges could include lower drug loading and entrapment efficiency, etc. The problems related to NP drug delivery which can be resolved with the aid of CDs are addressed in this review including poor drug loading into NPs, physical and chemical stability, specificity of drug target, pharmacokinetics and bioavailability related issues and modified drug release along with discussions on safety and efficacy aspects of CD in drug delivery. Especially this review is focused on the application of CDs in the elimination of difficulties in nanoparticulate formulation and improvement of their pharmaceutical and therapeutic performance. Additionally this manuscript provides insights into the theranostic applications of CDs.

## Cyclodextrin: overview

CDs are amphiphilic cyclic oligosaccharides containing at least six D-(+) glucopyranose units attached by α-(1, 4) glycosidic bonds.^28^ These are also known as cyclomaltodextrins or cycloamyloses. CDs are obtained from enzymatic degradation of starch from potato, corn and other sources. This was discovered by French scientist Villiers who isolated crystalline compound called ‘cellulosine’ in 1891.^29^ In following decade later, the role of glycosyltransferase from *Bacillus macerans* in production of CDs from starchwas demonstrated by Austrian microbiologist Schardinger. He identified naturally occurring CDs-alpha, beta and gamma which are referred to as Schardinger sugars. Moreover, he also identified β-CD as ‘cellulosine’ depicted by Villiers.^30^ Nowadays CDs are exploited for various applications in food, biotech, pharma, cosmetic and textile industry.^31-33^

Natural CDs α, β and γ-CDs with respectively 6, 7 and 8 glucopyranose units were included in the generally regarded as safe (GRAS) list of the USFDA for use as a food additive in 2004, 2001 and 2000 respectively. The recent regulatory status of natural CDs is revealed in Table 1. Two important CD derivatives hydroxypropyl-β-CD (HP-β-CD) and sulfobutylether-β-CD (SBE-β-CD) were cited in the FDA’s list of ‘Inactive Pharmaceutical Ingredients’ used in novel pharmaceutical applications along with natural CDs.^34^ The rational modifications of CD can be carried out for improving interaction with the biological membrane by increasing their lipophilicity, improving interaction of hydrophobic drugs with CD and allowing self-assembly of CD.

**Table 1:** Recent regulatory status of cyclodextrin

**Type of CD**	**Food Approval**			**Pharmacopoeia Monograph**		
	**US-FDA**	**Europe**	**Japan**	**USP/NF**	**European Pharmacopoeia**	**JPC**
α-CD	GRAS	Novel Food	NP	√	√	√
β-CD	GRAS	Food additive	NP	√	√	√
γ-CD	GRAS	Novel Food	NP	√	√	--

CD, Cylclodextrin; JPC, Japanese Pharmacopoeia; USP/NF, United state Pharmacopoeia/National Formulary, GRAS, generally regarded as safe; NP, natural product.

**Figure 1:**
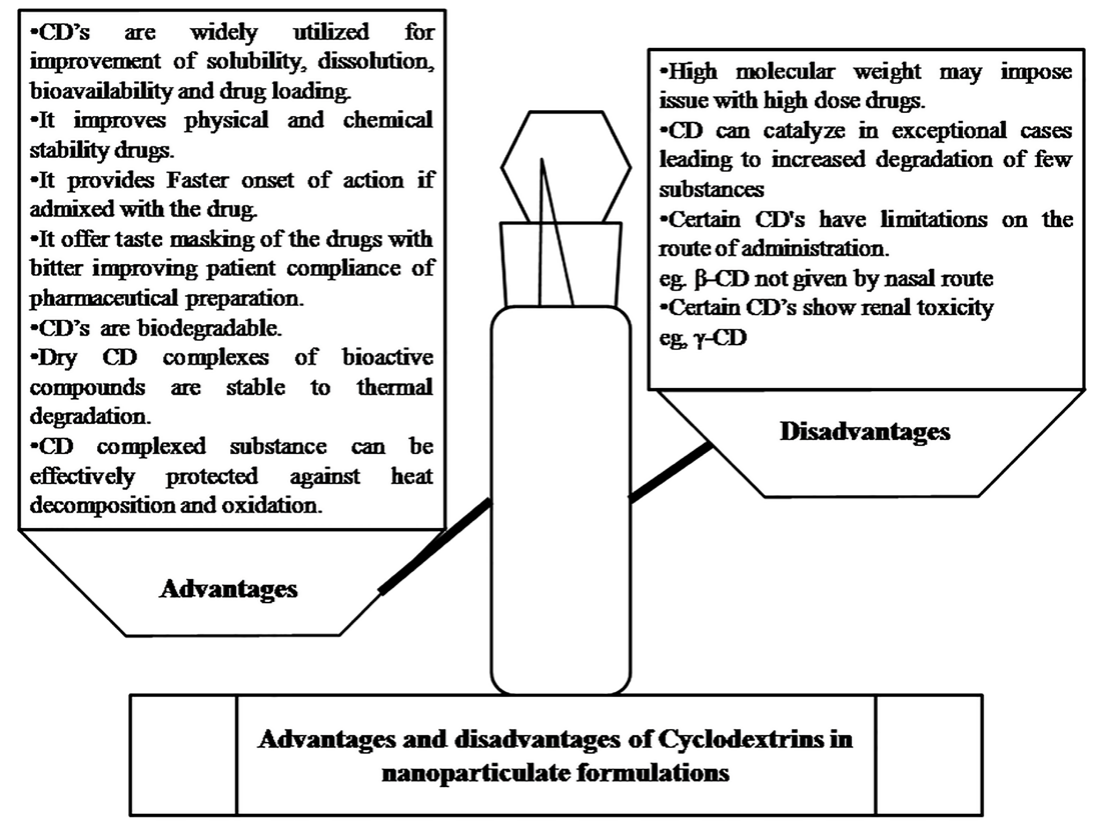
Advantages and disadvantages of cyclodextrins in nanoparticulate formulations.

CDs are commonly used for aqueous solubility enhancement of drug for oral delivery as well as in parenteral delivery as a result of their ability to form an inclusion complex with chemical moiety.^35,36^ They form complexes with a variety of molecules including organic, inorganic and organo-metallic compounds by so called molecular recognition phenomenon while their ability to form complexes with enantiomeric species is known as chiral recognition.^37^ The advantages and disadvantages of the CDs are listed in Figure 1.CDs are employed in the drug delivery due to their versatile potential related to drug permeability enhancement, bioavailability enhancement, improvement of safety and efficacy, improvement in drug and formulation stability, modified drug release and enhancement of drug loading, protein and peptide delivery, colon specific delivery, transdermal delivery, nasal delivery, pulmonary delivery^38-40^ and gene delivery.^41^

## Cyclodextrin-based nanoparticles

The CDs are widely studied and employed in solubility enhancement of poorly soluble drugs. CD can play a vital role in improving the performance of NP formulation. The natural CDs and their derivatives such as HP-β-CD and HP-γ-CD complexes or self-assemble in nanoscale aggregates in aqueous solutions.^42^ They can be exploited for improving drug loading, formulation stability, enhancing or improving absorption and bioavailability along with retained/improved safety and efficacy and modifying drug release through nanosystems.

The factors which affect *in vitro* characteristics of CD NPs including particle size, drug loading and release from formulation^43-45^ and stability^46^ are enlisted in Table 2.

**Table 2:** Factors affecting *in vitro* properties of CD nanoparticles

**Nanoparticle property**	**Influential Factors**
Particle size	CD substitution, Preparation technique
Drug loading and release	*a) CD related factors:* Concentration of CD, nature length of CD substitution, *b) Drug related factors:* Drug solubility (aqueous), Size and shape of guest molecule, Partition coefficient of drug, Molecular weight of drug, *c) Properties drug-CD complex:* Preparation technique, K_1:1_ association constant, Electrostatic interaction between CD & guest molecule
Stability	Surface charge, Steric factors (chain length and nature), Electrostatic interaction between host and guest molecule

CD, cyclodxtrin.

## Factors affecting in vitro properties of CD nanoparticles

### Nature, type and length of CD substitution

The natural CDs are modified by substituting them with different functional moieties with varied nature and length which provide cationic, anionic or nonionic amphiphilic nature to the CD. These modifications are necessary for improving the interaction of CDs with hydrophobic drugs which ultimately affects drug loading and drug release from NP formulation. The partially acylated β-CD regulate mean particle size of NPs and therefore the stability of the system.^47^ The alteration in drug loading, particulate size, entrapment efficiency and stability of formulation with modification on the primary and secondary face of β-CD are demonstrated in the literature.^48,49^ The correlation between the structure of the amphiphilic-CDs and their ability to form nanospheres shows that for the compounds that have hydrophilic lipophilic balance values are greater than 8.0 are water soluble, able to self organize in water to form nanospheres. Whereas, for the compounds with hydrophilic lipophilic balance values lower than 7.4 are soluble in organic solvent rendering the preparation of NPs by nanoprecipitation technique possible.

### Physicochemical characteristics of the drug

The most of drugs are either weak acids or weak bases. They vary in their physicochemical properties. The association constant of drug: CD, octanol: water partition coefficient, molecular weight, solubility, size and shape of drug (guest molecule) are considered to be influential factors for drug loading, entrapment efficiency and drug release from CD based NP.^50,51^ It is reported that in the study by Memisoglu-Bilensoy et al where hydrocortisone, testosterone and progesterone were drugs candidates for loading in CD based nanocapsule or nanosphere. Regardless of the technique of drug loading the progesterone a lipophilic drug with higher association constant and partition coefficient has reported having higher drug loading than other drugs.^52^

### Drug-CD complex properties

The complexation and electrostatic interaction between drug and CD can alter the pharmacokinetic profile of drug significantly. The effect of the presence of CD on Cmax, volume of distribution, mean residence time and renal clearance of drug was demonstrated by Charman et al.^53^ Drug-CD complex can be considered as molecular encapsulation. The CD molecule shields drug at least partly from an attack of the immediate external environment or reactive molecules. In this way, it may reduce or prevent drug degradation. The CD mimics enzyme catalysis or inhibition. The degree of stabilization/destabilization drug after the formation of inclusion complex depends mainly on the fraction of drug inside complex and rate of drug degradation inside complex.^54^

### Preparation and loading technique

The method and sequence of addition of the drug, CD and solvents varies with alteration in the preparation technique of CD-based NP. The assessment between the conventional method and emulsification method demonstrated that these methods leads to development to different colloidal structures, with different size and size distribution as well as different colloidal stabilities to nanocarrier. The residual amount of organic solvent may avoid coalescence of the colloidal system but their physiological effects should not overweigh the stability.^55^ High drug loading technique in which preformed steroidal drug: CD complexes were utilized along with the addition of drug during preparation have shown more drug loading in nanospheres than the conventional drug loading technique. The drug loading efficiency for nanocapsules is independent of techniques used for loading for steroidal drugs.^52^

## Methods of preparation for cyclodextrin based nanoparticles

Preparation technique dictates the formation of either nanospheres or nanocapsules. The drug loading can be carried out directly during preparation (conventional loading) or using previously formed drug: CD complexes (preloading) or a combination of both (High loading). The schematics of CD-NP preparation is depicted in the graphical abstract. Various methods of preparation of such CD based nano-systems are cited below.

### Polymer precipitation methods

In these techniques, polymer is precipitated with formation of either nanospheres or nanocapsules. These are easy and speedy production techniques with comparatively uniform size distribution. The mild solvents like ethanol, butanol or acetone may be selected for these techniques to avoid toxic residues generated from solvents like methylene chloride and chloroform, etc. Nonsurfactant NPs can be prepared in presence of CDs which can avoid complications associated with surfactants. Based on sequence and method of addition of drug and polymer, these preparation techniques can be divided as follows:

#### Solvent injection method

Hydrophobic components are dissolved in water-miscible organic solvent while hydrophilic components are dissolved in the aqueous phase. Organic phase injected into the aqueous phase, diffusion of organic phase into later phase leads to NPs formation.56 This method has an advantage that the based on the nature of drug and its solubility, it can be dissolved in either aqueous phase or organic phase.

#### Solvent evaporation method

In this method, a CD is dispersed in an organic solvent. Drug solution in the same solvent added to a CD dispersion. Then aqueous phase is added to this mixture and an organic solvent is evaporated to obtain nanospheres or nanocapsules which will be further freeze dried.^57^

#### Solvent diffusion

The solution of a drug in ethanol is slowly added to aqueous phase with a peristaltic pump with continuous stirring to form nanocrystals. The nanocrystal dispersion is clarified by centrifugation in the refrigerated condition which is then freeze dried.^58^

#### Inotropic gelation method

Sodium carboxy methyl β-CD (SCM- β-CD) or its mixture with pentasodium tripolyphosphate (TPP) in aqueous form was added to chitosan (CS) solution under magnetic stirring. The positively charged CS and negatively charged SCM- β-CD and/or TPP spontaneously react via inotropic gelation to form NPs.^59^

## Utility of Cyclodextrin functionalized nanoparticles

**Figure 2:**
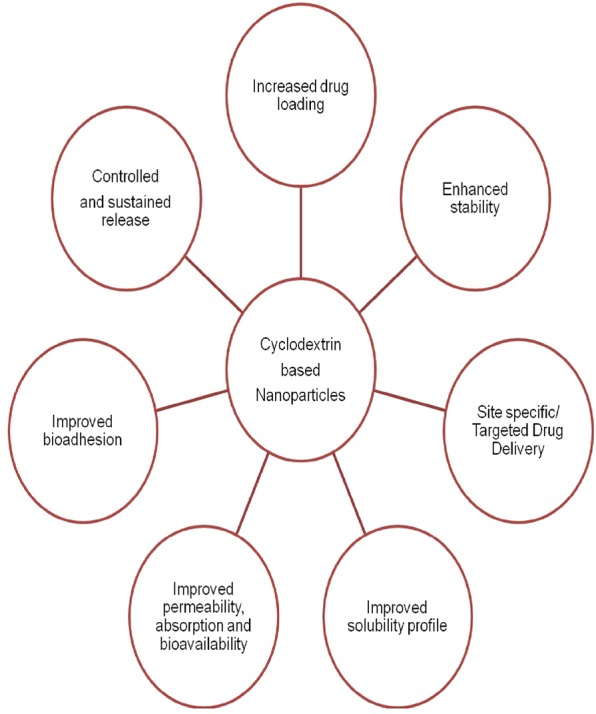
Applications of cyclodextrin based nanoparticles.

Nanoparticulate systems were employed for delivery of drugs as well as biomolecules. The obstacles in NP drug delivery can be overcome with the CD. The major avenues in the formulation of NP systems where CDs can be utilized to cause intervention for flawless drug delivery are depicted in Figure 2.

### Improvement of drug loading and entrapment efficiency

The higher drug loading is usually necessary in particulate systems for efficient utilization of the formulation system or carrier. The free energy of the host-guest complex is directly associated with binding constant and thus drug entrapment efficiency. The physicochemical properties of drug viz. molecular mass, aqueous solubility, partition coefficient and K_1:1_ association constant and loading technique mainly affect drug loading in CD functionalized NPs.^51^

Drug loading can be improved by substituted semi-synthetic CDs. Free paclitaxel in poly (anhydride) NPs has reported to show poor drug loading in comparison with its complex with HP-β-CD. The drug loading of paclitaxel was improved 500 folds with HP-β-CD against its polymeric NP.^60^ In an investigation by Yuan et al it was revealed that chitosan-graft-β–CD (CD-β–CS) has improved drug encapsulation of Ketoprofen by 1.36 against CS NPs. The order of entrapment efficiency was CS < CD9.6-β–CS < CD14-β–CS < CD20-β–CS, indicating significant improvement in entrapment with an increasing degree of substitution of CD on CS. This might be because that -CD could effectively load poorly water-soluble drugs into the cavity, additionally, this an increasing substitution shows increasing zeta potential.^61^ Drug loading of azole antifungal drugs was also improved when β-CD was modified on its primary and secondary face with substituent’s of varying chain length and bond type. It has been claimed that β-CD modified on the primary face with 6-carbon aliphatic chain with amide bond shows more drug loading than that of modification on the secondary face with ester bonds and 6-Carbon aliphatic chains.^62^ Encapsulation 6-coumarin in CS NPs was improved with various CDs.^63^ It is revealed that Erlotinib complex with β-CD sulfobutyl ethers (Captisol®) improve drug loading and entrapment efficiency in PLGA NPs along with improvement in solubility and cellular uptake in non small cell lung carcinoma.^64^

The ability of CDs increase drug loading and entrapment efficiency can be assigned to their ability to accommodate hydrophobic moieties.

### Stability enhancement

The stability of the nanoparticulate colloidal drug delivery system is a matter of concern to formulation scientists. The developed micro-particulate formulations shall be stable during shelf life. Zeta potential and stability of the nanoparticulate formulation is affected by type of excipients (usually by nature of polymers or lipid) and characteristics of alkyl chain in CD molecule.^46^

As discussed earlier nature and type of CD entail the characteristics of NPs, it was demonstrated that sulfated β-CD get associated with acylated CD to improve stability of nanospheres.65 In a recent study by Chen et al reports a kind of supramolecular assemblies constructed from two water-soluble and biocompatible saccharides, sulfonato-β-CD and CS shows adequate stability at temperature 10 to 70°C.^66^ β-CD has been utilized as capping agent and stabilizing agents for synthesis of copper NPs where it protects against oxidation of NPs and improves antibacterial activity.^67^ A report demonstrating improved stability of paclitaxel loaded solid lipid NPs has shown that HP-β-CD is more effective over hydroxy β-CD to control the particle size, polydispersity index and stability of NPs.^68^ The possible reason for stability of colloidal NPs using CDs may be balance created by hydrophilic and hydrophobic functions or it can be attributed to the steric interactions between alkyl chains in CD.

### Improvement in solubility, dissolution or bioavailability profile of drug

The hydrophilic affection of a drug is vital for drug loading in NPs and drug release from delivery system which consequently may affect the pharmacokinetics of the drug. Several approaches have been reported in literature for solubility enhancement of poorly soluble drugs. CD functionalized NPs are also widely employed for this purpose owing to the benefits offered by the CD discussed earlier.

It was reported that the bioavailability of raloxifene a selective estrogen receptor modulator, increased almost 2.6 folds by formation of CD/CS NPs using SBE-β-CD along with increasing drug solubility.^69^ It is revealed that CPT loading in CD NPs was higher as compared to polymeric NPs, with its solubilization and stabilization.^70^ A research report claims that there is increase in dissolution and bioavailability almost twice that of plain erlotinib (anticancer drug) when it is delivered in the form of erlotinib CD nanosponge complex. The improvement in solubility and dissolution of erlotinib was attributed to loss in crystallinity, particle reduction up to the molecular level and hydrogen bonding between the drug and CD-nanosponge.^71^ An interesting case reported regarding drug dutasteride which is insoluble in water (less than 0.038 ng/mL), shows 90% dissolution of the dutasteride from all the HP-β-CD nanostructures in dissolution media within nine minutes.^72^ In an investigation by Huarte et al where HP-β-CD, M-β-CD and SP-β-CD, at a concentration of 20% w/v, were capable to improve the aqueous solubility of CPT by factors of 24, 67 and 22, folds respectively. This indicates the significance of nature of CD having methyl groups in M-β-CD which enlarge the whole cavity of the molecule by extending the secondary hydroxyl side and narrowing the primary hydroxyl side of the cone and improves solubility to larger extent.^73^ The report indicating β-CD-curcumin NP complex has improved dissolution rate of curcumin by ten folds and improved permeability enhancement across skin.^74^

Insulin is a polypeptide hormone to control blood sugar level. The oral delivery of insulin has limitations due to its instability in under gastric environment. Carboxymethyl-β-CD-grafted CS (CMCD-g-CS) allows pH-triggered oral delivery of insulin at pH 7.4. The oral bioavailability insulin with this pH triggered grafted NPs as claimed by Song et al is far better than the bioavailability of plain insulin by subcutaneous or oral insulin.^75^ Alginate/trimethyl-CS NPs were reported for oral delivery of insulin containing cationic-β-CD, this also shows pH- trigger for simulated intestinal fluid (pH 6.8). It has shown to improve the permeability of insulin across Caco-2 cell layer.^76^ HP-β-CD complexed insulin was encapsulated in polymethacrylate based copolymer NPs. Insulin has shown to retain its activity with expected enhanced oral absorption.^77^ CMCD-g-CS has been additionally explored by Song et. al for oral delivery of other protein-bovine serum albumin showing its potential for delivery of proteinaceous candidates by oral route.^78^ CD-based star synthetic polymers having hydrophobic arms of acrylate group improved the cellular uptake of NPs. These acrylate groups can protonate at pH 6 which escapes the NPs from endocytotic vesicles and intracellular drug release acting as a platform for nanochemotherapeutics.^79^ A detailed account of effect of CDs on drug delivery through the biological membrane was reviewed by Loftsson et al.^80^ Docetaxel /SBE-β-CD/CS NPs showed capability in improving the small intestinal absorption and bioavailability by inhibiting the efflux of drug.^81^

### Site specific/targeted drug delivery

Delivery of optimal drug quantity safely to a specific site for effective therapeutics stems from the idea of minimizing risk to benefit ratio of the drug.^82^ CD-NPs have a potential of reducing drug toxicity with site specific/targeted delivery.

**Figure 3:**
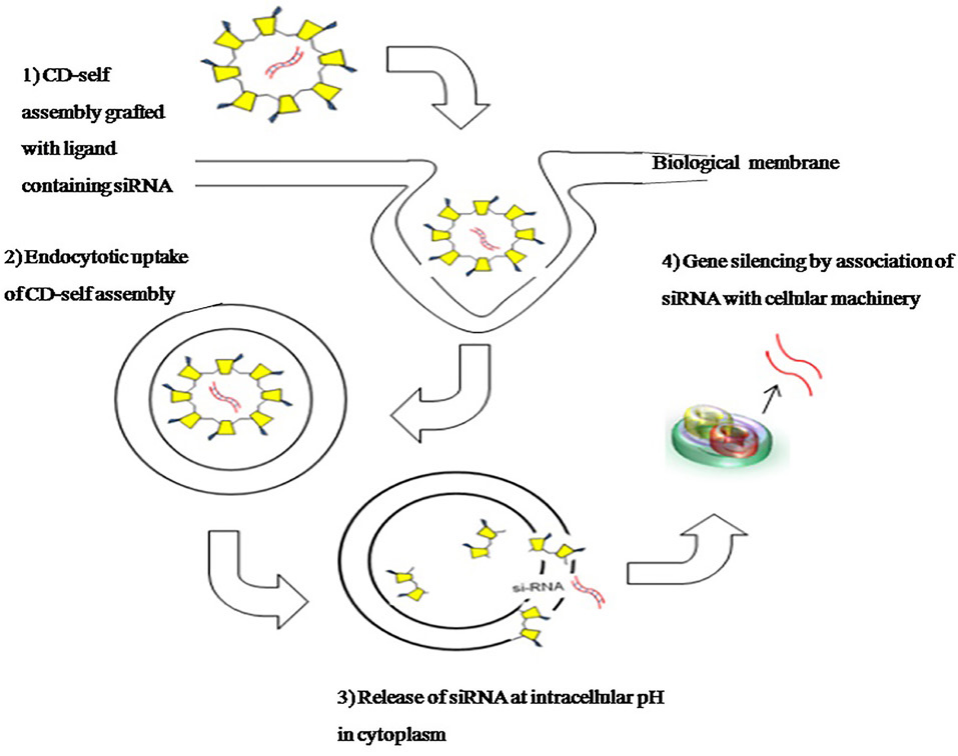
Schematic of CD as a carrier for intracellular drug delivery.

CD was utilized for siRNA delivery to target tumour cells the NP system consisting of CD, transferrin (targeting ligand) and polyethylene glycol.^83^ CDs utilized in development of NP for siRNA delivery works as polycation allow assembling with different size and types of nucleic acids through the electrostatic interactions. The formed nano-assemblies are resistant to the nuclease degradation. A reduction in both specific messenger RNA and protein RRM2 (M2 subunit of ribonucleotide reductase) was reported in phase I clinical trial with this NP system for the targeted treatment of solid tumours.^84^ The α–β CD dimer synthesized via click chemistry was utilized as a linker to connect hydrophobic and hydrophilic segment to form self assembled noncovalently connected micellar nanoassembly. Its application in ‘tumour triggered targeting’ was revealed through endocytosis experiments to confirm selective uptake of drug loaded micellar nanoassembly.^85^ The schematic of intracellular delivery biomolecule/drug using CD as a carrier is depicted in Figure 3.

Site specific delivery of indomethacin in polyethylamine-CD nanoassemblies to intestinal tissues resulted in lower gastric irritation with sustained drug release and mucoadhesive capabilities were reported.^86^ An interesting case of dual stimuli-responsive supramolecular polymeric NPs based on poly(α-CD) and acetal-modified β-CD-azobenzene reported by Dai et al. According the claims in this investigation, the drug release of methotrexate in faster in acidic conditions (pH 5.0), while ultraviolet (UV) irradiation shows burst release which slow down after the withdrawal of UV light indicating the promising application of CD based NPs in drug delivery to cancerous cells having acidic environment.^87^ Gene silencing with siRNA conjugated with β-CD was demonstrated by Malhotra et al. It is shown that modified β-CD has the ability of delivering RNA to cancer cells by simple complexation with polycationic lipids (lipoparticle) and by formulating inclusion complexes adamantyl-PEG-dianisamide with the CD-RNA conjugate NPs.^88^ The uptake mechanism of CD based NPs by tumour associated macrophage was demonstrated with murine glioma model which can used potentially to target malignant brain tumours.^89^ Lanthanide doped upconversion NPs absorbs near-infrared waves and convert them to UV light, additionally possess ability to penetrate deep tissues with the potential of bioimaging and treatment of diseases of deep tissues. Carboxymethyl-β-CD allows hydrophobic upconversion NPs to stay solution improving its functionality in cancer detection and treatment.^90^ It has been demonstrated in mice, significant proportion of melarsoprol from HP β-CD complex distributed into brain which could a positive avenue for its cerebral delivery in trypanosomiasis.^91^ Biotin modified β-CD gold NPs of paclitaxel were reported for pH- responsive targeted anticancer activity and lower toxicity to normal cells due to enhanced water solubility.^92^

Curcumin loaded-HP-γ-CD water soluble complex encapsulated in CS NPs not only enhance the passive targeting but also lead to high drug release within the cancer cells and enhance its efficacy.^93^ CDs modified with folic acid for targeted was disclosed by Hattori.^94^ Targeting efficiency of CDs modified with folic acid was exemplified with anticancer drug doxorubicin using folate receptor protein and Caco-2 cell layer model. Applications of CDs in targeting NPs were reviewed by Erdoar et al.^95^

### Mucoadhesion/bioadhesion characteristics

**Figure 4:**
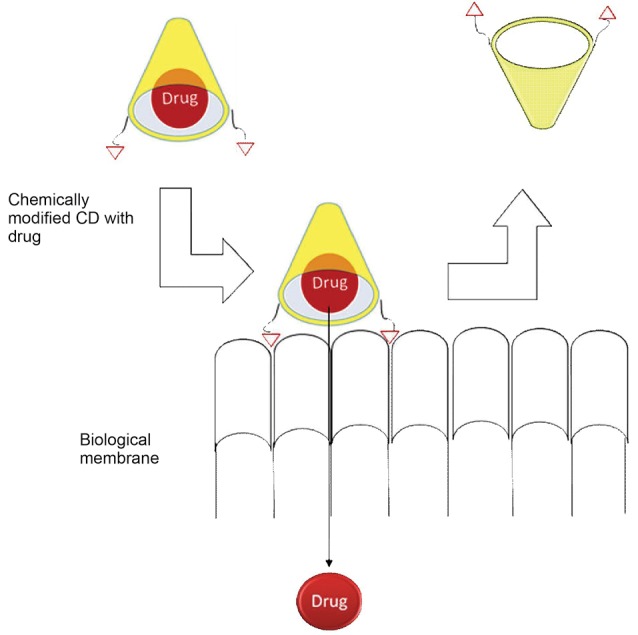
Schematic of chemically modified CD molecule for bio-adhesive/ muco-adhesive application.

Bioadhesive formulations are proposed to increase contact time with mucosa and in turn, improve drug absorption. CD-nanosystems possess potential of prolonging drug release through mucoadhesion/bioadhesion. The bioadhesive property of CD may facilitate to troubleshoot the drug permeability by increasing contact time of drug at surface of the mucosa. The chemical modification of CD can improve mucoadhesive/bioadhesive characteristics of NPs.^96,97^ The schematic of chemically modified CD carrier with bioadhesive or mucoadhesive abilities is illustrated in Figure 4.

SBE-β-CD/CS NPs for ocular drug delivery with prolonging the residence time of naringenin, which is useful for the treatment of age-related macular degeneration.^98^ Bioadhesive CS-coated CD-based supramolecular nanomicelles which improves the oral bioavailability of doxorubicin along with this they claimed to have a biodegradable and biocompatible nature too.^99^ A study showing a comparison between poly (anhydride) NPs of HP-β-CD and NPs coated with poly(ethylene glycol) 6000 for oral antigen/drug delivery has shown to possess cytoadesive nature but later shows more bioadhesion.^100^ β-CD modified mesoporous silica NPs with hydroxyl, amino, and thiol groups were reported in the literature. A comparison of their mucoadhesive properties and potential as a drug delivery system for superficial bladder cancer therapy revealed that thiol-functionalized NPs exhibit significantly higher mucoadhesivity on the urothelium as compared to the hydroxyl- and amino-functionalized NPs. ^101^

### Controlled and sustained drug delivery

Controlled and sustained drug delivery systems were developed for reducing the dosing frequency of drug. These systems provide a drug release over a predefined time period with the possibility of reducing the required dose and subsequently related side effects of the drug. Sustained or controlled release can be achieved with CD functionalized NPs. The drug Release rate and amount of drug released from CD functionalized NPs could be affected by preparation method, nature of CD, grafting or crosslinking agents, aqueous solubility of polymer and drug.^102-104^

**Figure 5:**
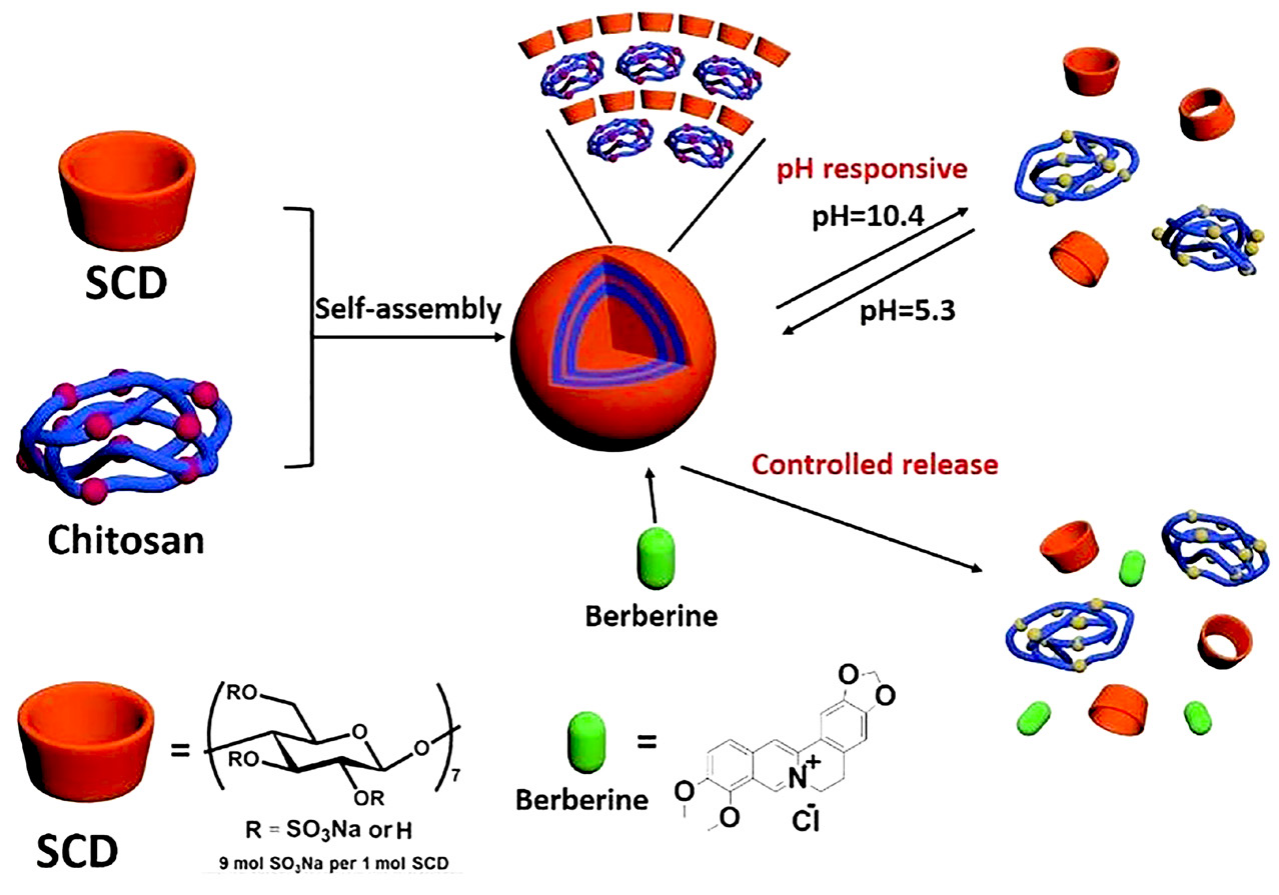
Schematic of chemically modified sufonato-CD (SCD) molecule for pH triggered drug delivery and controlled release of berberine. Reprinted (adapted) with permission from Chen et al.^66^

The biocompatible supramolecular assembly employing sulfonato-β-CD polysaccharides and CS as building blocks and different physiological pH as controlling method explored by for controlled release of berberine. The sulfonato-β-CD based carrier loaded with natural molecule berberine, allows drug release at intestinal pH and shows stability in the gastric environment. The schematics of sulfonato-β-CD/CS based supramolecular assembly is shown in Figure 5.^66^ An interesting case of cationic-β-CD/5-Flurouracil within alginate/CS nanoflowers has been presented by Lakkakula et al which provide sustained drug release in both acidic (pH 2.3) and basic (pH 7.4) conditions with significantly higher encapsulation efficiency and lower polydispersity index.^105^ Potent and long lasting (~22 hours) inhibitory activity on the pressor response of angiotensin-I was demonstrated through arterial blood pressure measurements in rats after administration of captopril-CD NPs.^106^ Docetaxel-loaded NPs assembled from β-CD/calixarene giant surfactants shows initial burst release followed by longer drug release for about 30 to 60 hours.^107^ Insert therapeutics-101, a linear CD-containing polymer conjugate of CPT formulated in NPs is under clinical trial showing prolonged release.^108^ Docetaxel loaded in heptakis (2-O-oligo (ethyleneoxide)-6-hexadecylthio-)-beta-CD has shown slow release allowing prolonged cell arrest in mitosis.109 It was demonstrated that copolymer containing β-CD can be utilized for self assembly hydrophobic drugs in nanovehicles to exhibit sustained release. Conjugates of poly (DL-lactide-co-glycolide) on amino CDs in NPs were reported for bovine serum albumin delivery showing triphasic release for 27 to 28 days.110 Injectable and biodegradable supramolecular hydrogel formed from two-level self-assembly of amphiphilic polymer methoxy poly(ethylene glycol)-b-poly (ε-caprolactone-co-1,4,8-trioxa[4.6]spiro-9-un-decanone) (mPECT) and polypseudorotaxane formed by α-CD and PEG blocks demonstrated release of NPs of ≈50 nm from hydrogel lasting for more than 3 weeks and subcutaneous biodegradability for ≈2 weeks.^111^

## Safety and efficacy aspects of cyclodextrin nanoparticles

The toxicity profiles of CDs depend on the route of administration. CDs are nontoxic by the oral route of administration. CDs forms complex with biliary salts and cholesterol in the gastrointestinal tract in a reversible manner. CDs administered by the intravenous route are hemolytic in action.^37^ However, some derivatives of CD like HP-β-CD is well tolerated and noncarcinogenic in some animal studies and some reports that it is unsuitable for use as a vehicle in oral (gavage) pre-clinical toxicology studies in the RccHan:WIST rat, owing to the effects on liver enzymes, urinary volume and microscopic findings in the kidneys.^112,113^ So, the use of HP-β-CD should be cautioned. However, the oral administration of β-CD nanosponges has indicated it’s the safety in animal studies allowing one to use the β-CD in drug delivery.^114^

The ability of CD to overcome a barrier in the formulation of poorly water-soluble drug CPT with improving its pharmacokinetics, pharmacodynamics and efficacy was demonstrated by CRLX101 formulation. It is composed of β-CD-poly (ethylene glycol) copolymer conjugated to CPT which appeared to be safe and effective in phase 1/2 clinical studies.^115^ Sugammadex is a selective relaxant binding agent with a modified γ-CD, and it is specifically designed to grab and encapsulate the aminosteroidal neuromuscular blocking agents such as rocuronium or vecuronium. Various clinical reports suggested the safety and efficacy of Sugammadex.^116^ Hydroxypropyl- and sulphobutyl ether CDs are recognized as safe and used in parenteral solutions. Although these more water-soluble derivatives of CD are well-tolerated α-CD, β-CD and methyl-CDs are considered toxic by parenteral route.^117^ The anticancer activity and acute toxicity of docetaxel-HP-β-CD is comparable to Taxotere, but CD based formulation not lead to hypersensitivity, which was observed in Taxotere treated group.^118^ It was demonstrated that α-methyl prednisolone conjugated CD polymer NPs significantly decrease arthritis score in animals which could be a safer and effective approach for rheumatoid arthritis treatment.^119^ Lower hemolytic activity and significantly low minimum inhibitory values were observed for azole antifungals in amphiphilic CD compared to their ethanolic solution indicating increased efficacy by nanospheres of CD drug inclusion complexes.^62^ Moreover, uses of modified CDs reduce/eliminate the need of surfactant in NP preparation further improving their intravenous safety.^120^ The promising improvements in antiviral activity of acyclovir by incorporating it with β-CD-poly(4-acryloyl morpholine) monoconjugate has been reported along with biocompatibility assays to prove safety.^121^ Nanosponges prepared by crosslinking of beta CDs with diphenyl carbonate were reported in recent studies encapsulating dexamethasone for ocular delivery which with demonstrated improved permeability and safety.^122^

These reports indicating safety and improved efficacy of CD could open new avenues in nanoparticulate drug delivery. One can precisely design and synthesize CD derivatives based on these previous studies. The recent regulatory status of CDs as discussed earlier can serve as evidence for its potential to be utilized it in drug and gene delivery. The safety evaluation and careful selection of CD derivatives to improve pharmaceutical characteristics can provide a unique opportunity in drug delivery.

## An account of recent advances in CDs for optimal drug delivery

CDs offer various benefits as discussed earlier in addition to this it possesses the ability to tailor-made the drug release to the target site. They possess the inherent ability to accommodate or associate with the wide variety of small molecules, proteins, peptide and aptamers. This can be achieved by careful selection of the proper type of CD or through the chemical modifications of CDs for the predetermined purpose of the delivery. The chemical modifications of CD are carried out for increasing interaction of CDs with biological membrane, enhancement of solubility and dissolution of the drug, accommodating hydrophobic and hydrophilic drugs, controlled or sustained release of the drug release, stimuli responsive drug release or for improving the stability of the formulation.^123^ Some recent case studies of the CDs in the optimal drug delivery of drugs, proteins, peptides and aptamers are reported in the Table 3.^124-140^ CDs can be moulded into various types of formulation for proving ease the drug delivery by diverse routes of administration.

**Table 3:** Account of recent paradigm CDs for drug Delivery

**Type of Cyclodextrin**	**Molecule and category**	**Route of administration**	**Formulation**	**Comments**	**Ref.**
2-hydroxy-propy-β-cyclodextrins (HPbetaCDs)	Itraconazole (Antifungal)	Pulmonary	Aerosol	Rapid absorption across the pulmonary epithelium compared to NP formulation of drug	^124^
Polymeric β-CDs	Ethionamide (Antitubercular)	Pulmonary	Nanoparticles	Drug incorporated following green protocol better for pulmonary administration	^125^
HP- β-CD	Prednisolone and Fludrocortisone acetate (Corticosteroid)	Pulmonary	Dry powder aerosol	CD promote dissolution and helps in permeation across a Calu-3 cell monolayer	^126^
Poly- β-CD	Ethionamide (antitubercular)	Pulmonary/ entdotracheal/ Intranasal	Nanoparticles	Empty Poly- β-CD has intrinsic antitubercular activity	^127^
Ethylenediamine derivative of​ β-​CD	Doxorubicin HCl (Anticancer)	Hep-G2 cell line study	Magnetic nanocomposites	NIR light −responsive controlled and targeted release	^128^
sulfobutylether- β-CD	Naringenin (antimicrobial Anticancer)	Ocular	sulfobutylether- β-​CD/chitosan NPs	CD/CS provided sustained release and improved bioavailability	^129^
µ-CD and γ-CD	Cyclosporin (Immunosupressant)	Ocular	Nanoparticles	γ -CD concentration has effects on aggregation but decreases particle size, safe for once or twice day administration	^130^
HP- β-​CD	Resveratrol (Antioxidant)	--	Polyvinylpyrrolidone-loaded resveratrol electrospinning nanofibers	Improved aqueous solubility of drug with sutained drug release	^131^
βCD or HPbCD	Glibenclamide (Hypoglycemic)	Oral	poly(anhydride) NPs	CDs allow higher payload in NPs with initial burst release followed by sustained drug release	^132^
βCD	Hyaluronic acid (anti-wrinkle effects and moisturizing agent)	Transdermal	quaternized βCD -grafted chitosan NPs	Quaternization of CD gives stable NPs, water retention capacity of hyaluronic acid is improved by crosslinking with polymer	^133^
HP- β-CD, Methyl- β-CD and Trimethyl-β-CD	Temoporfin (Photosensitizer for treatment of squamous cell carcinoma)	--	Drug-in-cyclodextrin-in-liposome (DCL) nanoconstructs.	trimethyl-β-CD-based DCL retains almost all drug and shows stability	^134^
HP-β-CD	Dolutegravir sodium (DTG)	Nose to brain delivery	CD-based NPs	CD-based NPs provided higher drug loading and 2.54 folds greater permeability of DTG compared to free drug	^135^
HP-β-CD	Efavirenz (Antiviral)	Intranasal	chitosan-grafted-HPβCD NPs	Chitosan and CD based NPs provide Sustained release, high brain targeted delivery and 4.76 times greater permeability than plain drug solution through porcine nasal mucosa.	^136^
HP-β-CD	Benznidazole (Antiparasitic)	--	Quatsomes and liposomes of	CD based nanofomulates shows better activity against Trypanosoma cruzi as compared to drug and its nanostructure lipid carries. It contributes to the solubility of drug in formulation.	^137^
β-CD	Curcumin (antioxidant, analgesic, anti-inflammatory and antiseptic)	--	βCD based nanosponge (CDNS)	Curcumin loaded CDNS shows the selective toxicity against cancerous cells and free CDNSs showed no toxicity	^138^
β-CD	Saporin (Cytotoxic protein)	--	βCD modified Circular bivalent aptamers (cb-apt)	Supramolecular ensemble exhibits high serum stability, molecular recognition ability and enhanced intracellular delivery efficiency.	^139^
β-CD	Doxorubicin (anticancer)	HER2- Cell line study	HApt aptamer-functionalized pH-sensitive β-CD-capped doxorubicin (DOX)-loaded mesoporous silica nanoparticles	βCD works as pH sensitive nanovalve for DOX release	^140^

CD, cyclodextrin.

Interested authors can read the recent review by Shelley and Babu on how CDs can be employed beneficially to boost the characteristics of the polymeric, magnetic, lipid, metallic and mesoporous NPs.^141^ A story of CDs for biomedical applications especially providing the details of supramolecular interactions by Mejia-Ariza et al is interesting to read.^142^ Add on to the accounts of CDs are provided by the reviews published in recent years giving up to date version of available opportunities for the researchers working on their use in the development of nanovesicles for diverse relevance in pharmaceutical and biomedical field.^143-145^

## Theranostic applications of cyclodextrins

Theranostics is an emerging therapeutic prototype that enables synchronized execution and accomplishment of diagnosis and therapeutics for the betterment of treatment. Therapeutic and diagnostic job in one delivery formulation tender combined approach, theranostic agents facilitate disease diagnosis, therapy, and instantaneous monitoring of advancement treatment and efficacy, all with one pharmaceutical agent.^146^ Theranostics is extensively explored in the diagnosis and treatment cancer due to unique rewards offered by stimuli-responsive nanosoldiers in malignant pathology, allowing the nanocarrier to respond specifically to the pathological ‘triggers’ such as pH, enzyme, redox microenvironment, temperature and small molecules for smart delivery of molecules to tumour.^147^

CD-NPs are employed as the ultimate carrier for the delivery of therapeutically vital compounds. Ample variety of available CDs makes it suitable carrier or transporter to accommodate compounds of diverse nature for biomedical and pharmaceutical applications. The ability of CDs offering modification functional groups on its surface to allow encompassing of versatile molecules mainly by host guest interactions and electrostatic interactions. CDs has emerged it as appropriate candidate for theranostics applications due to its flexibility offering chemical modification which can act as (a) a core for development of highly branched star polymer (b) a bridge or linker for different polymer block (c) a gatekeeper for the inorganic NPs. CDs are graced with biocompatible characteristics and catalytic attributes for well-located, feasible, stimuli responsive smart delivery of molecules.^148^ Representative case studies of theranostic applications of CD are specified in Table 4.^149-163^

**Table 4:** Theranostic applications of cyclodextrins

**Type of Cyclodextrin**	**Name of synthesized polymer**	**Molecule**	**Use**	**Comments**	**Ref**
Β-CD	folic acid-functionalized poly(β-cyclodextrin-co-pentetic acid)	Gadolinium (III) oxide	Contrast agents for magnetic resonance imaging	Poly-CD shell could significantly enhance biosafety reducing the toxicity gadolinium (III) oxide	^149^
Β-CD and 6-thio- β-CD	β-CD functionalized Polydopamide- Fe_3_O_4_ magnetic nanoparticles	Diclofenac	Nanovehicle for targeted drug delivery	CD improved drug loading and allowed slow release of drug for more than 75 hrs	^150^
Methyl-β-CD	Methyl-β-CD-quaternary ammonium chitosan conjugate (QA-Ch-MCD)	Dexamethasone	Mucoadvesive carrier	QA-Ch-MCD with drug shows particle size 2.7±0.4 nm showing higher stability constant and complexation efficiency against methyl-β-CD	^151^
β-CD	poly(p-phenylene-β-cyclodextrin)-graft-poly(ethylene glycol)	--	Cell imaging and radiotherapy	Glycoconjugation, provides more effective targeting and imaging with β-CD groups compared to unconjugated polymer	^152^
β-CD	β-CD-Maleic Anhydride N-Isopropylacrylamide	Curcumin Doxorubicin Hydrochloride	Fluorescence imaging, cancer cell-targeting, dual stimuli (pH and temperature) response and dual drug delivery	CD allows loading and release of hydrpphillic and hydrophobic components in nanoconjugates, Simultaneous loading and release of Doxorubicin and Curcumin. Endocytosis monitored by fluorescence and targeted cancer cell death in vitro manifested by significant tumor regression in vivo	^153^
Mono(6-amino-6-deoxy)- β-CD	Reactive oxygen species (ROS)- cleavable thioketal linker (TK) bridged β-CD dimmer and ROS non-responsive alkyl linker bridged β-CD dimmer	Camptothecin	ROS responsive for selective killing of tumor and the real-time tracking of drug release	Modified thioketal derivative of CD permit ROS resposnsives for optimal characteristic of nanosystem	^154^
HP-β-CD	HP-β-CD functionalized Fe3O4	Doxirubicin	pH/NIR responsive drug release and magnetic resonance/near infrared fluorescence imaging-guided synergetic chemo/photothermal therapy of tumor	HP-β-CD functionalized Fe3O4 responsible for pH dependent drug release behavior,	^155^
β-CD	β-CD NPs linked to tetrafluoroterephthalonitrile	Doxorubicin	pH-triggered release allow cancer targeting, with controlled release characterized by very fast cell uptake kinetics due to sugar-receptor mediated endocytosis	β-CD derivative allows thermal stability and high water dispersibility	^156^
β-CD	Star polymer composed of β-CD core and poly(2-(dimethylamino) ethyl methacrylate) (PDMAEMA) arms	Reduced Glutathione	MRI contrast agent and drug delivery	β-CD based star polymer provides higher drug association and better stability in serum solutions against linear polymers	^157^
β-CD	βCD -{poly(lactide)-poly(2-(d imethylamino) ethyl methacrylate)-poly[oligo(2-ethyl-2-oxazoline)methacrylate]}_21_ unimolecular micelles	Doxorubicin	Dual-functionalization for CT imaging and drug delivery	CD provides hydrophobic core for drug loading, stearic stabilization of CD based polymer provides gold NPs	^158^
Carboxy-methylated β-CD	CM-β-CD was grafted onto Fe_3_O_4_ on fluorescent dye-conjugated silica layer-folic acid	Retinoic acid	Smart theranostic candidate for simultaneous fluorescence imaging, magnetic manipulation, cancer cell-targeting and hydrophobic drug delivery.	Fe_3_O_4_ is encapsulated within a shell of SiO2 that ensures biocompatibility of the nanocomposite and a host for fluorescent dye, cancer-targeting ligand (folic acid), and a hydrophobic β-CD).	^159^
β –CD	β -CD-grafted polyethylenimine (CP)	siRNA	siRNA targeting the M2 isoform of the glycolytic enzyme pyruvate kinase (PKM2)	CP provides positive charge for loading of siRNA through electrostatic interaction and enables effective endosomal escape of siRNA	^160^
β –CD	β -CD PEG, biotin and b-CD surface-functionalized AuNP and PEG, biotin and rhodamine B linked β-CD surface-functionalized AuNP	Paclitaxel	Higher affinity to cancer cells such as HeLa, A549, and MG63 indicating its role in the diagnosis and therapy of the cancer cells	PEG used as a solvated antifouling shell, biotin as a cancer-specific targeting ligand, β -CD as a drug pocket	^161^
mono-6-thio β –CD	Polydopamine (PDA)-coated magnetic nanoparticles functionalized with mono-6-thio-β-cyclodextrin (SH-βCD)	Doxorubicin	Combined chemo- and photothermal therapy (CT-PTT) of liver cancer	Prepared NPs are nontoxic and shows higher drug loading	^162^
CM-β –CD	Antibody modified polypyrrole CD	Doxorubicin	photoacoustic imaging-guided chemo-photothermal therapy for thyroid cancer	Three-stimuli-controlled drug delivery, including the enzyme-sensitive, pH-sensitive and photothermal-sensitive drug release	^163^

CD, cyclodextrin; NP, nanoparticle; CT imaging, computed tomography imaging.

## Conclusion and Future Perspective

The accumulated pieces evidence in last two decades conclusively demonstrates that CD can play versatile role in the improvement of nanoparticulate or nanovesicular drug delivery. The CD functionalization could be valuable for increasing drug loading, improving solubility, stability, permeability, absorption, bioavailability and modifying drug release with retaining safety and efficacy. Recently reported acetylated α-CD materials were used for pH-modulated hydrolysis and pH-triggered drug delivery of paclitaxel exploring the new generation of nanocarriers.^164^ Further chemical modifications of CDs and formulation studies are needed to exploit their applications in site specific controlled delivery of drugs through NPs which can be seen from the typical examples indicated in Table 5.^165-173^

**Table 5:** Summary of CD nanoparticles used for targeted and site specific delivery

**CD-Type used**	**Drug/molecule**	**Formulation type**	**Target site/Cell line**	**Disease/ Disorder/Application**	**Ref.**
PEGylated CD	SiRNA	Injectable preparation	Prostate	Cancer	^165^
Β-CD-bearing Gold-GlycoNP	Methotrexate	Gold NP	cDNA clone for the human galectin-3	Cancer	^166^
Β-CD-poly(5-amido iso-phthalic acid)	Docetaxel	Magnetic NP	HeLa and MDA-MB-231 cancerous cell line cells	Cancer	^167^
lactoferrin-modified β-CD	Near-infrared fluorescent dye IR-775 chloride	Injectable NP	Brain	Neurological disease and as diagnostic reagents	^168^
Sulfobutyl ether β-CD	Naringenin	β-CD/chitosan NP	Ocular	Topical ophthalmic delivery	^98^
Polycationic amphiphilic cyclodextrin	Plasmid pCMVLuc VR1216 (6934bp) encoding luciferase	Nanocomplexes	HeLa or HepG2 cells.	Gene delivery	^169^
α-CD	doxorubicin hydrochloride	Supramolecular Hydrogels Based on PEG-PLA-Block Copolymer Micelles and α-CD	HeLa cells	Controlled release in Cancer	^170^
β-CD	Ibuprofen	Magnetic NP double coated with β-CD chitosan	HEPG-2, MCF-7 and BEL-1	Magnetically targeted and controlled release	^171^
β-CD	Reservatrol	pH-sensitive nanoparticles loaded into microbubbles	Hepato-carcinoma (H22) cells	pH responsiveness, targeted treatment, and ultrasound tumor imaging	^172^
β-CD	Doxorubicin	NP	HeLa cells	Cancer	^173^

CD, cyclodextrin; NP, nanoparticle.

The CD allows NPs to be tuned for tailor-made needs of drug delivery and theranostics through the chemical modifications demonstrating the scope for the researchers from the chemical, biomedical and pharmaceutical field to focus on the utilization of CD in nanoparticulate drug delivery.

Along with the issues discussed under this review it attracts our attention to the recent reports indicating the intervention of CD derivatives in the treatment of various diseases. CRLX 101 a β-CD based NP formulation has reported intervention for the treatment of various types of carcinoma including non small cell lung cancer, ovarian cancer, gastroesophageal cancer, etc.^174^ The reports showing safety of α-CD along with modest reduction in small low density lipoproteins, and an improvement in glucose related parameters and use of HP-β-CD for treatment of Niemann-Pick disease, type C1 provide as opportunity for synergistic delivery of the molecules used in the treatment of these diseases.^175,176^ Additionally, HP-β-CD is known to reduce local irritation antiviral intravenous formulation of letermovir.^177^ These studies unlock new avenues in the research of drug delivery science for better management opportunities of diseases and their side effects.

## Ethical Issues

Not applicable.

## Conflict of Interest

Authors declare no conflict of interest in this study.

## Acknowledgments

The authors are grateful Dr. J.N. Sangshetti, Mr. Vishal A. Chakkarwar and Mr. Sachin R. Patil for their suggestions and revision of manuscript.
